# Outbreak investigation of *Serratia marcescens* neurosurgical site infections associated with a contaminated shaving razors

**DOI:** 10.1186/s13756-020-00725-6

**Published:** 2020-05-12

**Authors:** Eun Jin Kim, Wan Beom Park, Jung-Ki Yoon, Won-Sang Cho, Su Jung Kim, Young Rok Oh, Kang Il Jun, Chang Kyung Kang, Pyeong Gyun Choe, Jong-Il Kim, Eun Hwa Choi, Myoung Don Oh, Nam Joong Kim

**Affiliations:** 1 Center for Infection Control and Prevention, Seoul National University Hospital, Seoul, South Korea; 2 Department of Internal Medicine, Seoul National University College of Medicine, 101 Daehak-ro, Jongno-gu, Seoul, 03080 South Korea; 3 Department of Translational Medicine, Seoul National University College of Medicine, Seoul, South Korea; 4 Department of Neurosurgery, Seoul National University College of Medicine, Seoul, South Korea; 5 Genomic Medicine Institute, Medical Research Center, Seoul National University, Seoul, South Korea; 6 Department of Pediatrics, Seoul National University College of Medicine, Seoul, South Korea

**Keywords:** Surgical site infection, *Serratia marcescens*, Disease outbreak, Whole-genome sequencing

## Abstract

**Background:**

Surgical site infection (SSI) is the most common healthcare-associated infection. We report an outbreak of neurosurgical site infections caused by *Serratia marcescens* after craniotomy in a tertiary care hospital.

**Methods:**

Between August 6 and 21, 2018, five cases of early-onset SSI caused by *S. marcescens* after craniotomy were recorded in a 1786-bed tertiary care hospital. Cultures were collected from potential environmental sources and healthcare workers. Whole-genome sequencing (WGS) was used to investigate the genetic relationships among *S. marcescens* isolates.

**Results:**

The outbreak involved five patients; *S. marcescens* was isolated from the cerebrospinal fluid, pus, tissue, and blood samples from these patients. *S. marcescens* was also isolated from shaving razors and brushes. All *S. marcescens* isolates from the infected patients and razors showed the same resistance patterns on antibiotic-susceptibility tests. WGS revealed close clustering among four of five isolates from the patients and among three of four isolates from the razors. No additional patient developed *S. marcescens* infection after we stopped using the razors for scalp shaving.

**Conclusions:**

We report an outbreak of neurosurgical site infections after craniotomy, which was associated with shaving razors contaminated by *S. marcescens*. Shaving scalps with razors should be avoided to prevent SSI.

## Background

A recent prevalence study found surgical site infections (SSIs) to be the most common healthcare-associated infection (HAI), accounting for 31% of all HAIs among hospitalized patients [1, 2]. SSI, a serious complication of surgery, is associated with significant morbidity, mortality, and health care costs [3, 4]. *Serratia marcescens* is a gram-negative bacteria widely distributed in the environment. It is a well-known etiologic agent of HAIs, including bacteremia, urinary tract infection, meningitis, pneumonia, and SSIs [5–7].

We report an outbreak of neurosurgical site infections by *S. marcescens* after craniotomy in a tertiary care hospital. We describe the outbreak investigation and the measures taken to control it.

## Methods

### Background and epidemiological investigation

The outbreak occurred in a 1,786-bed tertiary care hospital in Seoul, South Korea. Between August 6 and 21, 2018, five cases of early-onset SSI caused by *S. marcescens* after craniotomy were confirmed using the 2018 National Healthcare Safety Network surveillance definitions [8].

The Infection Control Team has performed surveillance of SSIs after craniotomy since 2007 and observed a significant increase in the number of patients with neurosurgical site infection. *S. marcescens* had hitherto been an uncommon pathogen causing SSI, and the incidence of neurosurgical site infection by *S. marcescens* significantly increased during the outbreak. As soon as an SSI outbreak was suspected, the team monitored the practices and interviewed personnel in the operating room, neurosurgical ward, and barbershop. Based on information from observation and interviews, environmental samples were obtained for culture. A total of 22 samples were collected from water sources, scrub sinks, surfaces, and shaving razors in the operation room. Another 296 samples were collected from surgical instruments, and 39 nasal samples were collected from doctors, nurses, and other healthcare workers who were associated with neurosurgical operations and the hospital barbershop. An additional 30 samples were collected from the equipment used for preoperative shaving, including razors, brushes, solid soap for shaving, and other tools in the barbershop.

All environmental samples were immersed in 3 mL brain heart infusion (BHI) broth and cultured aerobically for 24 h at 35 °C ± 2 °C. The BHI was inoculated onto blood agar plates and MacConkey agar and incubated for 24 h at 35 °C ± 2 °C. Bacteria were identified using matrix-assisted laser desorption/ionization-time of flight (MALDI-TOF), and antimicrobial susceptibility tests were performed using either VITEK 2 or Microscan Walkaway 96 plus instruments.

### Molecular biological analysis (microbiological investigation/genotyping)

Whole-genome sequencing (WGS) was performed with nine *S. marcescens* isolates, including five strains from patients with SSI and four from razors. Genomic DNA extracted using a QIAamp DNA Mini Kit (QIAGEN, Hilden, Germany) was quantified using a Qubit®2.0 instrument (Life Technologies, Burlington, Canada), and sequencing libraries were generated using an Illumina TruSeq DNA PCR-Free Kit (15036187; Illumina). Briefly, the samples were normalized to 1 μg DNA and sheared by sonication with a Covaris S220. AMPure XP beads were used for cleanup and size selection, and adapters were then ligated. The fragment sizes for all libraries were measured using a 2100 Bioanalyzer (Agilent Technologies, Palo Alto, CA, USA), and quantitative polymerase chain reaction (qPCR) was performed on a LightCycler® 480 System (Roche, CA, USA) with a KAPA Library Quantification Kit (KK4854, KAPA Biosystems). Whole-genome sequencing was performed on an Illumina Nextseq500 instrument with 300-cycle reagent. To filter adapter and low quality sequences, all reads were trimmed using fastp (version 0.13.1) with default parameters [9]. Kraken2, a taxonomic classification system using exact k-mer matches [10], was used to detect the reference genome and *S. marcescens* subsp. *marcescens* Db11 was found to have the closest genome to those of the isolates. The trimmed reads were mapped to the reference sequence using BWA-mem [11], and single nucleotide polymorphisms (SNPs) were detected using SAMtools and bcftools [12] using default parameters except the following: minimum mapping quality score 30, minimum base quality score 20, and ploidy 1. For comparison, using the same process, we processed two *S. marcescens* genome data, PRJEB28358 and PRJEB27112, publically available in the European Nucleotide Archive (ENA) database consisting of 19 and 17 isolates, derived from the outbreaks in Germany and India, respectively [13, 14]. The core regions were defined as the genomic positions having depths from all 47 isolates ranging between mean depth ± 2 standard deviations. Phylogenetic analysis was conducted using MEGA-X (version 10.0.5) [15] with SNPs only in core regions. The distances between isolates were computed with the maximum composite likelihood method [16], and the phylogenetic tree was built by an unweighted pair group with arithmetic mean (UPGMA) method. The test of phylogeny was performed using the bootstrap test (number of bootstrap = 100).

### Statistical analysis

The prevalence of SSI in the pre-outbreak and outbreak periods was compared by chi-square tests using IBM SPSS Statistics for Windows, version 23.0 (IBM Corp., Armonk, NY).

## Results

### Outbreak investigation

Surveillance of SSIs following craniotomy since 2007 revealed a prevalence of 1.82% (165/9,058) until July 2018. Between August 6 and 21, 2018, five cases (11.63%) out of 43 patients who underwent craniotomy developed neurosurgical SSI due to *S. marcescens*. The prevalence of neurosurgical site infection increased significantly compared to that in the pre-outbreak period (*p = 0.003*). Between January 2014 and July 2018, the prevalence of SSI following craniotomy was 1.03% (45/4,384). During this period, the most common etiologic microorganism was *Staphylococcus aureus* (22.2%), followed by coagulase-negative staphylococcus (21.6%). *S. marcescens* accounted for 8.3% of etiologic microorganisms. Considering the significant increase in the prevalence of SSI and proportion of *S. marcescens*, we decided to start an outbreak investigation. Between August 6 and 21, 2018, there was another case of SSI after craniotomy which was attributed to *Staphylococcus aureus.* Four cases of SSI were monomicrobial infections caused by *S. marcescens*, and one case was a polymicrobial infection caused by *S. marcescens* and *Klebsiella aerogenes*.

The epidemiological and clinical characteristics of the patients are summarized in Table 1. *S. marcescens* was isolated from cerebrospinal fluid (three patients), pus (four patients), tissue (one patient), and blood (one patient). Three of the five patients (60%) were female, and the mean age of the patients was 39 years (range 11–76 years). The mean interval between surgery and positive culture was 7.2 days (range 3–11 days). These patients were located in different wards and their operations were performed by different surgeons. All patients had undergone preoperative scalp shaving at the barbershop in the study hospital. Among the 387 surveillance samples (22 from the water sink and surface of operating rooms, 296 from surgical instruments, 30 from the barbershop, and 39 from healthcare workers), *S. marcescens* was isolated from two shaving brushes and four razors that had been used to shave patients’ hair before surgery. Microorganisms other than *S. marcescens* were detected in 53 samples collected from environment and healthcare workers. (Table 2) All patients were treated with antibiotics, and some underwent wound debridement and/or external ventricular drainage.

**Table 1:** Epidemiological and clinical characteristics of outbreak patients with SSI caused by *Serratia marcescens* after craniotomy

Patient	Age/sex	Date of surgery	Date of isolation	Operative procedures	Isolates	Scalp shaving using razors	Management of surgical site infection
1	76/female	8/6/2018	8/9/2018	Craniotomy, Occipital artery to posterior inferior cerebellar artery bypass	CSF, Blood	Yes	Antibiotics, EVD
2	66/female	8/6/2018	8/13/2018	Craniotomy, tumor removal	CSF, Pus	Yes	Antibiotics, EVD
3	11/female	8/6/2018	8/14/2018	Craniotomy, Encephaloduroarterio synangiosis	Wound, Pus	Yes	Antibiotics, wound debridement
4	14/male	8/10/2018	8/21/2018	Craniotomy, Encephaloduroarterio synangiosis	Pus	Yes	Antibiotics, wound debridement
5	29/male	8/21/2018	8/28/2018	Craniotomy, tumor removal	CSF, Pus, Tissue	Yes	Antibiotics, EVD

CSF, cerebrospinal fluid; EVD, extraventricular drain

**Table 2:** Surveillance cultures from environmental samples and healthcare workers

	Environment			Healthcare workers (*n* = 39)	Total (*n* = 387)
	Surface of sink, operating room (*n* = 22)	Surgical instruments (*n* = 296)	Tools for shaving or in the barbershop (*n* = 30)		
Positive culture (%)	7 (31.8)	10 (3.4)	25 (83.3)	17 (43.6)	59 (15.2)
*S. aureus*, n (%)			2 (6.7)	2 (5.1)	4 (1.0)
CNS, n (%)	2 (9.1)	9 (3.1)	6 (20.0)		17 (4.4)
Bacillus spp., n (%)	5 (22.7)		1 (3.3)		6 (1.6)
Enterococcus spp., n (%)			1 (3.3)		1 (0.3)
Micrococcus spp., n (%)		1 (0.3)			1 (0.3)
Klebsiella spp., n (%)				14^a^(35.9)	14 (3.6)
Enterobacter spp., n (%)			3 (10.0)		3 (0.7)
Raoultella spp. n (%)			3 (10.0)		3 (0.7)
Escherichia spp. n (%)				1 (2.6)	1 (0.3)
Pseudomonas spp.,, n (%)			3 (10.0)		3 (0.7)
*S. marcescens*, n (%)			6 (20.0)		6 (1.6)

*CNS* Coagulase negative staphylococcus, ^a^ 13 *Klebsiella aerogenes* and 1 *Klebsiella pneumoniae*

### Implementation of infection control measures

Upon detection of *S. marcescens* on the razors, we stopped using the razors for scalp shaving in the barbershop, ward, and operating room. Contamination of operating rooms, instruments, or specific procedures such as wound dressing, and bacterial colonization among health care workers were checked while investigating the outbreak, but there was no common source of *S. marcescens* infection other than brushes and razors in the barbershop. We recommended that trained doctors or nurses shave scalps using disposable clippers for surgery when shaving was necessary. Infection Control team nurses performed rounds frequently to enforce standardized skin preparation procedures and the use of clippers. After implementation of these infection control measures, no further patients developed *S. marcescens* infections.

### Antibiotic susceptibility and molecular biological analysis

The *S. marcescens* isolates from infected patients and razors shared the same resistance pattern in antibiotic-susceptibility testing. The isolates were resistant to amoxicillin/clavulanic acid and nitrofurantoin and susceptible to piperacillin/tazobactam, ceftriaxone, cefepime, ertapenem, imipenem, meropenem, gentamicin, ciprofloxacin, and trimethoprim/sulfamethoxazole.

**Fig. 1:**
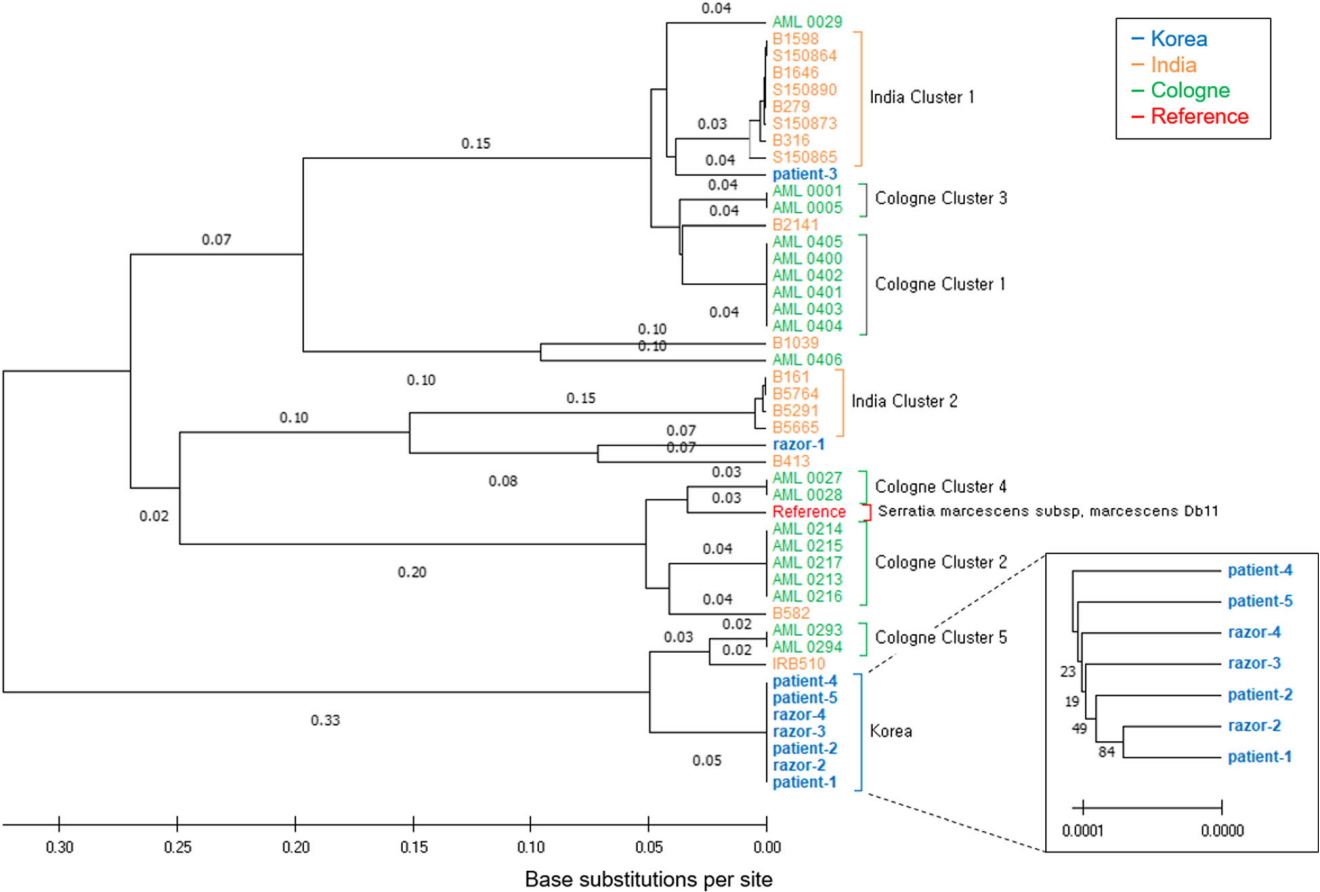
The Phylogenetic tree of *S. marcescens* isolates across three outbreaks in Korea, India, and Germany. An UPGMA phylogenetic tree of *S. marcescens* isolates from the three outbreaks was generated using the 420,357 core SNP positions detected. The tree reveals the tight clustering of isolates from four patients and three razors in Korea (blue). The other brackets (orange for India, green for Cologne in Germany) annotate clusters described in the previous studies, and all clusters have the same isolates as observed in this study. The small box shows an ~ 100 times enlarged subtree of the clustered isolates. The scale bar and branch lengths are in the units of the number of base substitutions per site. The percentage of replicate trees in which the associated taxa clustered tighter in the bootstrap test (100 replicates) is shown close to the branches in the small box

WGS was performed for nine *S. marcescens* isolates, five strains from patients with SSI, and four from razors. The mean sequencing depth of the nine isolates in this study was 220× (185–244×) and the number of SNPs in the core regions was 142,316 on average. With WGS data of 36 *S. marcescens* isolates derived from the outbreaks in Germany and India, a total 420,357 bp of core regions with SNPs were used to build the phylogenetic tree. Phylogenetic analysis using WGS revealed close clustering between four of five patient isolates and three of four razor isolates (Fig. 1). *S. marcescens* isolated from patients and razors were expected to be of the same strain, but there was a considerable distance genetically between the strains isolated from patient 3, razor 1, and others.

## Discussion

Neurosurgical site infection occurs in 2.2–4.7% of patients after craniotomy [17]. SSI after craniotomy has been associated with increased morbidity, risk of reoperation, neurological sequelae, and mortality. A meta-analysis revealed that the risk factors for SSI included CSF leakage, CSF drainage, operation duration, venous sinus entry, and American Society of Anesthesiologists score [18]. In the current report, we found that contaminated brushes and razors were the sources of the outbreak and that the inadequate shaving technique might cause serious neurosurgical site infections.

It is common practice to shave a patient’s hair around the surgical area. Hair removal has benefits for surgical wound visualization, dressing application, and avoidance of potential annoyance due to hair. Furthermore, many surgeons believe that hair removal is helpful for the prevention of SSI. However, the results of randomized controlled trials revealed no differences in SSIs between patients with and without hair removal before surgery [19]. In the context of neurosurgery, the prevalence of SSIs also does not differ with scalp shaving [20–23]. Tokimura et al. conducted a retrospective study of 632 patients who underwent cranial surgery without head shaving, of which only 7 (1.1%) developed postoperative infections [20]. Horgan et al. and Ratanalert et al. conducted randomized studies with 20 patients and 225 procedures respectively [22, 23]. In Horgans’ trial, 10 patients had been assigned to the non-shaved group, and there was no infection in both groups. In Ratanalert’s trial, 89 procedures had been allocated to the non-shaved group, and the rates of SSI were 3.37 and 5.88%, respectively. (*p* > 0.05) Further, a systematic review including 18 studies on the effect of preoperative scalp shaving on SSI showed that preoperative scalp shaving was unnecessary for the asepsis of surgical sites [21]. However, as those studies are underpowered, more research is needed to obtain conclusive evidence. Moreover, the guidelines from the Centers for Diseases Control and Prevention and the World Health Organization recommend that hair not be removed unless it interferes with the surgical procedure [24, 25]. If it is necessary to remove hair, clipping rather than shaving is recommended in light of infection control [19]. In the present study hospital, scalp shaving was performed by barbers in the barbershop. Shaving razors were reused between patients, which eventually resulted in an outbreak of SSI. The infection control team prohibited the use of razors to shave scalps and recommended disposable clippers if scalp shaving was necessary. Following the implementation of these recommendations, no further patients have developed SSI by *S. marcescens*.

Typing methods for discriminating different bacterial isolates of the same species are essential tools in outbreak investigation. A typing method must have the discriminatory power to distinguish related and unrelated isolates. These methods are classified as phenotypic or molecular. Phenotypic methods include antibiograms, serotyping, and biotyping, while molecular methods include pulse-field gel electrophoresis, multi-locus sequencing typing, and variable number tandem repeat typing. Following improvements in sequencing technologies, whole-genome sequencing (WGS) is a highly discriminative tool that has become the gold standard for bacterial typing, as it can define the complete genomic structure of a pathogen [26, 27]. In this study, we applied WGS to investigate the relatedness of *S. marcescens* isolates, finding close genetic relationships between four patient and three razor isolates. The strain isolated from patient 3 did not show close genetic similarity with the other strains; therefore the *S. marcescens* infection in patient 3 should be considered as irrelevant to the *S. marcescens* outbreak.

We obtained nasal samples rather than samples from the hands for bacterial culture. Considering that bacterial colonizations in the hand sample is a more meaningful indicator of SSI than that in nasal sample, it would be better to consider hands as a site of surveillance culture.

The epidemiological and microbiological evidence indicated that contaminated razors and brushes in the barbershop were the sources of the outbreak. The outbreak ended after stopping preoperative shaving in the barbershop and instead using disposable surgical clippers. Several studies have also reported outbreaks associated with contaminated razors or brushes [28, 29]. The findings of the present report have implications for clinicians and infection preventionists. First, inadequate shaving technique may cause severe SSIs. Second, the use of razors to shave scalps is not recommended because it is associated with an increased incidence of SSI. Third, regular surveillance for SSIs may be beneficial for early outbreak detection. Infection control practitioners can play an important role in screening and preventing outbreaks of SSI based on the prevalence of SSI and the distribution of pathogens.

## Conclusions

We reported an outbreak of neurosurgical site infections associated with shaving razors contaminated by *S. marcescens*. To prevent SSIs, razors should not be used to shave scalps. As recommended in several guidelines, proper hair removal using disposal clippers before surgery is important for the prevention of SSIs.

## Data Availability

WGS data were deposited at Short Read Archive (assessment number PRJNA609822). Other data generated or analyzed during this study are included in this published article.

## Abbreviations

SSI: Surgical site infection
WGS: Whole-genome sequencing
HAI: Healthcare-associated infection
BHI: Brain heart infusion
